# NEWS, NEWS2, and qSOFA accuracy in predicting sepsis-related mortality in acute myeloid leukemia: a retrospective single-center analysis

**DOI:** 10.1097/j.pbj.0000000000000266

**Published:** 2024-10-14

**Authors:** Ana M. Meireles, Leonardo M. Moço, Cláudia S. Moreira, Gil P. Brás, Ana E. Santo, Mário Mariz

**Affiliations:** a Department of Haematology and Bone Marrow Transplantation, Portuguese Oncology Institute of Porto (IPO Porto), Porto, Portugal; b Clinical Oncology Group, IPO Porto Research Centre (CI-IPOP), Portuguese Oncology Institute of Porto (IPO Porto), Porto, Portugal

**Keywords:** febrile neutropenia, acute myeloid leukemia, early warning signs

## Abstract

Acute myeloid leukemia (AML) treated with intensive chemotherapy carries a high risk of severe infection. The development of reliable assessment tools to promptly identify patients at risk of developing critical illness is essential to prevent delays in intensive care unit (ICU) admission. This study evaluated the accuracy of quick Sequential Organ Failure Assessment (qSOFA) score, National Early Warning Score (NEWS), and NEWS2 score in predicting ICU admission and sepsis-related mortality in this population. A retrospective analysis was conducted, including 365 episodes of febrile neutropenia in 126 patients. The results showed that all three scores—qSOFA, NEWS, and NEWS2—demonstrated good accuracy for all outcomes, with area under the receiver-operating characteristic curve values for sepsis-related mortality of 0.812, 0.858, and 0.848, respectively. In addition, the scores exhibited excellent accuracy in predicting ICU admission and the composite outcome of ICU admission or sepsis-related mortality. To our knowledge, this is the first study to evaluate the accuracy of NEWS in a population of patients with AML who did not undergo stem cell transplantation. These findings suggest that NEWS and NEWS2 are effective tools for identifying patients with AML at high risk of clinical deterioration during febrile neutropenia, supporting their use in clinical practice.

## Introduction

Acute myeloid leukemia (AML) treated with intensive chemotherapy carries a high risk of severe infections.^1^ A meta-analysis of 73,213 critically ill patients suggests that earlier admission to the intensive care unit (ICU) reduces mortality, particularly in patients with cancer.^2^ Early warning scores (EWS) are tools designed to identify hospitalized patients at risk of clinical deterioration who may require additional support beyond standard care. While there are several available scoring systems, Nagarajah et al^3^ suggest that the National Early Warning Score (NEWS and NEWS2) may be the most appropriate for oncologic patients. However, the validity of these scores in the context of hematological patients remains underexplored. Thus, our study aimed to evaluate the accuracy of NEWS, NEWS2, and quick Sequential Organ Failure Assessment (qSOFA) in predicting ICU admission and/or sepsis-related mortality in AML patients with febrile neutropenia (FN) during intensive chemotherapy.

## Methods

This was a retrospective, single-center, observational, cohort study. We included all consecutive cases of febrile neutropenia in patients aged 18 years or older undergoing intensive chemotherapy for acute myeloid leukemia (AML) between January 2012 and June 2022. Patients who underwent hematopoietic stem cell transplantation or were diagnosed with acute promyelocytic leukemia were excluded, as the latter is associated with a lower risk of sepsis. Data were collected from the electronic medical records at IPO Porto.

The variables collected included age, sex, comorbidities, treatment regimens, infection site, identified microbiological agents, vital signs, major organ dysfunction in patients admitted to the ICU, and clinical outcomes. Data extraction was performed independently by two researchers using a prespecified data collection form.

The clinical scores were calculated on the day when the patient's condition was at its worst during febrile neutropenia, based on vital signs: respiratory rate ≥22/min, altered mental status (Glasgow Coma Scale <15), systolic blood pressure ≤100 mmHg, fever (>38.0°C), or tachycardia (>100 bpm). The primary outcome was sepsis-related mortality, while secondary outcomes included ICU admission and a composite variable incorporating both mortality and ICU admission.

Continuous variables were described using median and interquartile range. The accuracy of the scores was assessed by calculating the area under the receiver-operating characteristic curve (AUROC). Score performance was classified as “good” with an AUROC between 0.8 and 0.9 and as “excellent” with an AUROC between 0.9 and 1. Statistical analyses were performed using SPSS version 26.

## Results

A total of 365 consecutive FN episodes in 123 patients met the study criteria: 62.7% (229/365) were female, with a median age at admission of 57 years [47–64] and a median Charlson Comorbidity Index of 1 [0–2].

In terms of treatment phase, 50.1% (183/365) of the episodes occurred during induction therapy and 49.9% (182/365) during consolidation. Sepsis-related mortality among all FN episodes was 4.4%, and the ICU admission rate was 7.1%. An etiological agent was identified in 40% (145/365) of the episodes: 30.3% (44/145) involved gram-positive bacteria, 65.5% (95/145) involved gram-negative bacteria, and 4.1% (6/145) were associated with a positive Aspergillus antigen. The characteristics of neutropenia are summarized in Table 1.

**Table 1:** Description of the febrile neutropenia episodes included in the cohort.

Febrile neutropenia feature variables	N = 365
Treatment phase	
Induction	183 (50.1%)
Consolidation	182 (49.9%)
Infection site	
Unknown	207 (56.7%)
Digestive	51 (14.0%)
Respiratory	37 (10.1%)
Skin/soft tissues	24 (6.6%)
Central venous catheter	17 (4.7%)
Mouth	7 (1.9%)
Anorectal	14 (3.8%)
Urinary	8 (2.2%)
Identified microbiological agent	145 (39.7%)
Gram-positive	44 (30.3%)
Gram-negative	95 (65.5%)
*Aspergillus spp.* antigen	6 (4.1%)
Early warning signs	
NEWS	3 [2–5]
NEWS2	3 [2–5]
qSOFA	0 [0–1]
Missing	28 (7.6%)
Early warning signs among intensive care unit–admitted patients	26 (7.1%)
NEWS	11 (8–12)
NEWS2	11 (8–12)
qSOFA	1 (1–2)
0 or 1	11 (42.3%)
2 or 3	15 (57.7%)
Major dysfunction among intensive care unit–admitted patients	
Vasoactive drugs	13 (50.0%)
Mechanical ventilation	15 (57.7%)
Renal replacement	5 (19.2%)
Death due to sepsis	16 (25.0%)

NEWS, National Early Warning Score; NEWS2, National Early Warning Score 2; qSOFA, quick Sequential Organ Failure Assessment.

**Figure 1.**
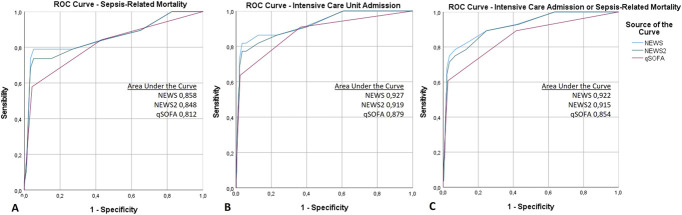
ROC curves of the accuracy of the qSOFA, NEWS, and NEWS2 scores in predicting (A) sepsis-related mortality, (B) intensive care unit admission, and (C) composite outcome of sepsis-related death and intensive care unit admission. NEWS, National Early Warning Score; NEWS2, National Early Warning Score 2; qSOFA, quick Sequential Organ Failure Assessment; ROC, receiver-operating characteristic curve.

The median NEWS and NEWS2 scores were 3 [2–5], and the median qSOFA score was 0 [0–1]. All scores demonstrated good accuracy in predicting sepsis-related mortality and excellent accuracy in predicting ICU admission as well as the composite outcome of sepsis-related death or ICU admission, as shown in Figure 1.

Regarding the performance of the scores in predicting outcomes in febrile neutropenic patients, for sepsis-related mortality, NEWS (AUC = 0.858) and NEWS2 (AUC = 0.848) demonstrated good predictive accuracy, while qSOFA (AUC = 0.812) performed slightly lower but still within an acceptable range. For ICU admission, NEWS (AUC = 0.927) and NEWS2 (AUC = 0.919) exhibited excellent discrimination, while qSOFA (AUC = 0.879) had a good performance. For the combined outcome of ICU admission and sepsis-related mortality, NEWS (AUC = 0.922) and NEWS2 (AUC = 0.915) again outperformed qSOFA (AUC = 0.854), showing excellent predictive accuracy (Fig. 1).

## Discussion

Our results underscore the superior predictive accuracy of the NEWS and NEWS2 scores compared with qSOFA, particularly in predicting ICU admission and the composite outcome of ICU admission and sepsis-related mortality. Although all scores performed well, NEWS and NEWS2 consistently demonstrated better performance, especially in critical situations where intensive care was required.

Several retrospective studies have highlighted the utility of EWS in patients at risk of sepsis, including those with hematological malignancies such as AML. Frairia et al retrospectively evaluated 418 episodes of FN in patients with AML undergoing intensive treatment, including those who had undergone allogeneic stem cell transplantation. They assessed the accuracy of NEWS and qSOFA in predicting death from sepsis within 24 hours of admission, with findings showing AUROC values of 0.924 for NEWS and 0.884 for qSOFA, indicating high accuracy for both scores, though NEWS demonstrated a slightly higher predictive value.^4^ Lappalainen et al conducted a study including 355 episodes of FN in patients with AML receiving first-line intensive treatment, exploring the association between qSOFA scores and mortality. They found that a qSOFA score of 2 or higher was significantly associated with increased mortality.^5^ Another retrospective study, involving 334 patients with AML (34% of whom had undergone allogeneic transplantation), also validated the predictive capabilities of NEWS and qSOFA for mortality during FN, with AUROC values of 0.894 for NEWS and 0.827 for qSOFA.^6^ Collectively, these studies highlight the potential utility of both NEWS and qSOFA scores in managing AML patients with FN, aiding clinicians in identifying high-risk patients who may benefit from more intensive monitoring and interventions.

This study has several strengths, including its homogeneous, single-center population that excluded patients who had undergone allogeneic transplantation, providing a focused and consistent sample. With a reasonable sample size of 365 FN episodes, it offers substantial data for statistically relevant analysis. In addition, it addresses a population for which there is limited available evidence, contributing valuable insights into the field. Moreover, the study is based on cost-effective scoring systems that can be easily applied in clinical practice by both nursing and medical teams, enhancing its practical relevance and potential for widespread implementation.

However, the retrospective design introduces potential biases. The lack of sequential evaluation of the scores may have limited the ability to capture a more dynamic understanding of patient progression over time. In addition, assessment of the day of clinical deterioration was subject to some degree of subjectivity. These limitations suggest that while the study offers important insights, further prospective research incorporating sequential score evaluations would be beneficial to confirm and expand upon these findings.

In conclusion, NEWS and NEWS2 demonstrated excellent accuracy in predicting sepsis-related mortality and ICU admission in patients with AML undergoing intensive chemotherapy and presenting with FN. These findings support the utility of these scores as effective tools in clinical practice for the early identification of high-risk patients, which may ultimately lead to improved patient outcomes.

## References

[1] Halpern AB Culakova E Walter RB Lyman GH. Association of risk factors, mortality, and care costs of adults with acute myeloid leukemia with admission to the intensive care unit. JAMA Oncol. 2017;3:374–381. 27832254 10.1001/jamaoncol.2016.4858 PMC5344736

[2] Hourmant Y Mailloux A Valade S Lemiale V Azoulay E Darmon M. Impact of early ICU admission on outcome of critically ill and critically ill cancer patients: a systematic review and meta-analysis. J Crit Care. 2021;61:82–88. 33157309 10.1016/j.jcrc.2020.10.008

[3] Nagarajah S Krzyzanowska MK Murphy T. Early warning scores and their application in the inpatient oncology settings. JCO Oncol Pract. 2022;18:465–473. 34995083 10.1200/OP.21.00532

[4] Frairia C Nicolino B Secreto C . Validation of national early warning score and quick sequential (sepsis-related) organ failure assessment in acute myeloid leukaemia patients treated with intensive chemotherapy. Eur J Haematol. 2023;110:696–705. 36919878 10.1111/ejh.13952

[5] Lappalainen M Hämäläinen S Romppanen T . Febrile neutropenia in patients with acute myeloid leukemia: outcome in relation to qSOFA score, C-reactive protein, and blood culture findings. Eur J Haematol. 2020;105:731–740. 32740997 10.1111/ejh.13500

[6] Nicolino B Vitolo U Audisio E . National early warning score (NEWS) and quick sequential (Sepsis-Related) organ failure assessment (qSOFA) validation in AML patient during febrile neutropenia. Blood. 2017;130(Suppl 1):5016.

